# Evaluation of Cardiovascular Activity and Emotional Experience in Healthcare Workers (HCWs) Operating in COVID-19 Wards

**DOI:** 10.3390/jcm11247372

**Published:** 2022-12-12

**Authors:** Ermanno Vitale, Veronica Filetti, Francesca Vella, Paola Senia, Lucia Rapisarda, Serena Matera, Claudia Lombardo, Denis Vinnikov, Venerando Rapisarda, Caterina Ledda

**Affiliations:** 1 Occupational Medicine, Department of Clinical and Experimental Medicine, University of Catania, 95123 Catania, Italy; 2 Human Anatomy and Histology, Department of Biomedical and Biotechnology Sciences, University of Catania, 95123 Catania, Italy; 3 Faculty of Medicine and Healthcare, Al-Farabi Kazakh National University, Almaty 050040, Kazakhstan; 4 Department of Occupational Health Risks Laboratory, Peoples’ Friendship University of Russia (RUDN University), 117198 Moscow, Russia

**Keywords:** COVID-19, healthcare workers, cardiovascular activity, emotional experience

## Abstract

The new 2019 coronavirus or SARS-CoV-2 has been the first biological agent to generate, in this millennium, such a global health emergency as to determine the adoption of public health measures. During this sanitary emergency, the emotional experience of healthcare workers (HCWs) has been hugely tested by several factors. In fact, HCWs have been exposed to greatly tiring physical, psychological and social conditions. The authors investigated the cardiocirculatory activity of a group of HCWs as well as how they perceived stress while working in COVID-19 wards. In particular, every HCW underwent a medical check, an electrocardiographic base exam, systolic and diastolic pressure measurement, and cardio frequency measurement. Furthermore, each HCW was provided with a cardiac Holter device (HoC) and a pressure Holter (Hop). Some psychological factors were considered in order to quantify the stress perceived by each HCW while at work through the administration of two questionnaires: the “Social Stigma towards Patients due to COVID Scale (SSPCS)” and the “Professional Quality of Life Scale (ProQOL)”. The HoC and HoP analysis results for HCWs working in COVID-19 OU wards showed significant variations in cardiocirculatory activity. From the analysis of the SSPCS questionnaire answers, it is clear that all of them showed a sense of duty towards their patients. The analysis of the ProQOL questionnaire answers showed that the prevailing attitude is fear; however, HCWs did not absolutely discriminate against those who had COVID-19 nor did they refuse to help those in need. Continuous monitoring of these employees, also carried out through occupational medicine surveillance, allows for the detection of critical conditions and the implementation of actions aimed at preventing chronic processes.

## 1. Introduction

Since the COVID-19 emergency started, healthcare workers (HCWs) have been on the frontline coping with the pandemic, being exposed not only to infection risks but also to a remarkable emotional overload [1,2].

During this pandemic, the emotional experience of HCWs has been hugely tested by several factors: lack of adequate individual respiratory airway protection devices (PPDs) [3,4], especially in the early phase of the pandemic; COVID-19 patients’ care being carried out in critical conditions (ISS COVID-19, n.2/2020 Rev. Report); and the health system being under great pressure due to staff and structural shortages [4,5,6,7,8].

Further factors have included working in wards other than their own; dealing with critical conditions which would require greater experience (mainly for new graduates or specializing undergraduates); continuing to work despite having been in contact with COVID-19 patients; and fear of being infected as well as fear for their own relatives [3,9,10,11,12].

These are just a few examples which prove how all HCWs are presently exposed to greatly tiring physical, psychological and social conditions [11]. The so-called “Vaccine day”, 27 December 2020, was the day which marked the official start of the vaccination campaign against COVID-19 all over Europe. In Italy, the vaccine distribution started on 31 December [13]. The arrival of the vaccine allowed a significant reduction in hospitalizations, intensive care entries and deaths [12,14,15].

The literature dedicated to work-related stress has widely confirmed how the healthcare system is itself characterized by physical and psycho-social risk factors, which are closely linked to work organization and to the workers’ safety and health, including factors such as shifts, availability (including night work), urgency–emergency management, lack of personnel, daily coping with extremely harsh situations, and potential risks of verbal and/or physical aggression [16,17,18]. All these may contribute to mental strain, resulting in huge stress reactions, with possible short/longer-term psychological consequences [19,20].

Several previous studies have dealt with the consequences of work-related stress and anxious–depressive disorders on cardiac activity and vascular tone [21,22,23]. Epidemiological data shows that chronic stress predicts the onset of coronary heart disease (CHD) [23,24,25]. Employees who experience work-related stress and individuals who are socially isolated or lonely have an increased risk of a first CHD event [26,27,28,29,30]. In addition, short-term emotional stress can act as a trigger of cardiac events among individuals with advanced atherosclerosis [26,27,28,29,30,31].

In this study, the authors investigated the cardiocirculatory activity of a group of HCWs as well as how they perceived stress while working in COVID-19 wards.

## 2. Materials and Methods

### 2.1. Study Design

This observational study analyzed the relations between cardiovascular activity and emotional experience in a group of HCWs operating in COVID-19 Operative Units in a specified pandemic time period (April–June 2021).

### 2.2. Participants

A group of 30 (100%) healthy HCWs working afternoon shifts (02:00–08:00 pm) were recruited on a voluntary basis. The survey was conducted in April–June 2021 in an emergency hospital in southern Italy.

The inclusion criteria were working in COVID-19 Operative Units (COVID-OUs), no medications taken, no detected pathologies, and non-smoking habits. The exclusion criteria were not working in COVID-19 OUs, taking medications, known pathologies, and smoking habits.

A control group of 30 (100%) healthy HCWs (matching 1:1) also working afternoon shifts (02:00–08:00 pm) but not in COVID-19 OUs (no COVID-19 OU) were recruited, all of which were similar with respect to anthropological features and working seniority. Each HCW was given a diary in which to report the activities of each day and when each activity was carried out, including meal times; bed and wake-up times; times of preparation and entry into a COVID-19 OU; and the onset of symptoms, physical efforts and/or emotional changes throughout the day.

From the diary examination, the presence of events (favorable/unfavorable) other than the routine ones caused the registration of that day to be ruled out.

### 2.3. Medical Examinations

Every HCW underwent a medical check, with anamnestic confirmation of the HCW’s family history of cardiovascular diseases, an electrocardiographic base exam (ECG), systolic (SAP) and diastolic (DAP) pressure measurements, and measurement of cardio frequency (CF) with an electrocardiograph (ESAOTE P8000, Genova, Italy).

Venous blood was sampled (10 mL) to carry out routine tests, also taking into account the lipid profile of every subject.

Each HCW was provided with a cardiac Holter device (HoC) (Spiderview & Synescope v3.10, MICROPORT^®^, Clamart, France) and a pressure Holter (Hop) (Agilis Mini™, MICROPORT^®^, Clamart, France).

HoC and Hop measurements were performed continuously for 8 h:2 h before the shift (T1); throughout the whole 4 h work shift (T2); and 2 h after the work shift ended (T3).

Before starting registration, each HCW was asked to not do physical activity and drink coffee in the 2 h prior; just a light meal and drinking water were allowed.

### 2.4. Psychological Examinations

Some psychological factors were considered to quantify the stress perceived by each HCW while at work. The following questionnaires were administered: the “Social Stigma towards Patients due to COVID Scale (SSPCS)” [16,18,32]; and the “Professional Quality of Life Scale (ProQOL)” [33,34].

The SSPCS has been translated and developed and used to assess stigma [16]. This questionnaire was adapted from the instrument described by See et al. (2011) [35]. This instrument consists of 12 items that have been revised from the previous version [16]. The SSPCS has 3 subscale sections: discrimination (items 1–4) (i.e., “you feel it is not worth serving people who are most at risk of contracting the COVID-19”); nonacceptance (items 5–8) (i.e., “if a colleague or one of their relatives has frequent contact or works with people who have contracted the virus, I would advise them to change department or job”); fear (items 9–12) (i.e., “the best way to prevent COVID-19 infection is to avoid any contact with someone who have contracted COVID-19”). The 12 items are rated on a 4-point response scale, where 1 = definitely no, 2 = no, 3 = yes, 4 = definitely yes; higher scores indicated a more positive professional attitude [18,32].

The ProQOL was invented by Stamm (2005) [33] and later re-adapted for Italian users by Palestini et al. (2009) [34]. The Italian version of the Professional Quality of Life Scale (ProQOL) aims to measure the professional quality of life of accident and emergency workers based on three dimensions: assessment of risk of compassion fatigue (CF), potential for compassion satisfaction (CS), and risk of burnout (BO). Higher scores on the CF subscale (C: 7 items) indicate the respondent is at higher risk. Higher scores on the CS subscale (CS: 8 items) indicate the respondent is experiencing higher satisfaction with their ability to provide care (i.e., caregiving is an energy-enhancing experience, increased self-efficacy). Higher scores on the BO subscale (BO: 7 items) indicate the individual is at risk of experiencing burnout symptoms (e.g., “I felt I was experiencing the same trauma as the person I was treating”). Items are rated on a 5-point response scale, where 1 = never, 2 = rarely, 3 = sometimes, 4 = often, and 5 = very often. The alpha coefficient was 0.9 for CS, 0.82 for CF, and 0.82 for BO.

### 2.5. Statistical Analysis

Statistical analysis was carried out with SPSS software (IBM Corp., SPSS Statistics for Windows, Version 23.0., Armonk, NY, USA). The collected data was included in a database built ad hoc. The descriptive statistics were used to characterize the groups of subjects in the study and the association between the different variables was analyzed with a chi-square test (X2) or Fisher’s exact test and Student’s *t*-test. Furthermore, one-way ANOVA was applied to compare the variables for all three time points (T1, T2, T3). Statistical significance was set at *p* < 0.05.

## 3. Results

The sample under examination (workers in COVID-OUs) was made up of 18 men (60%) and 12 women (40%). The control group also had the same gender composition. Analysis of all 30 HCWs (workers in COVID-OU) revealed that the mean age of the sample was rather low (41.1 ± 7.7 years), with a working seniority of 10.5 ± 6.4 years. Similarly, the control group showed a mean age of 40.3 ± 8.1 years, and a working seniority of 10.1 ± 5.6 years. Of the HCWs (workers in COVID-OUs), 30% (n = 9) were physicians, 50% (n = 15) nurses, and 20% (n = 6) healthcare assistants, all with full-time contracts. The control group consisted of 33% (n = 10) physicians, 50% (n = 15) nurses, and 17% (n = 5) healthcare assistants, all with full-time contracts. All HCWs had been duly vaccinated with two doses of the Comirnaty Vaccine (BioNTech, Pfizer, Monza, Italy) in January–February 2021. Table 1 shows the main characteristics of the sample.

After comparing HCWs working in COVID-19 OUs with those in the control group, there were no statistically significant differences.

The antibody titer against SARS-CoV-2, detected at least 30 days after administering the second vaccine dose, showed protective values: 291.8 ± 304.7 AU/L and 314.8 ± 371.6 AU/L in the cases and the controls, respectively.

All healthcare operators in the COVID-19 OU group and the control ones had normal ECGs, and their hematochemical tests were also normal, including the lipid profiles (data not reported). In the same way, average basal levels (T1) of SAP, DAP and CF fell within normal ranges [36]. Results obtained from HoC and HoP analysis showed significant variations in cardiocirculatory activity (Table 2). In detail, mean values of SAP and DAP recorded at T1 were significantly higher in the T2 recordings (while working in COVID-19 wards) and they went back to basal levels at T3, although in a statistically significant way. CF values significantly increased from T1 through T2, then returned to basal levels at T3, in a statistically significant way. SAP, DAP and CF at T2 and T3 were significantly greater in COVID-19 OU HCWs than in the control group. Instead, no statistically significant differences were observed at T1, T2 and T3 for SAP, DAP and CF in the control HCW group. These results were also confirmed by the statistical analysis that compared the variables for all three time points. Indeed, mean values of SAP, DAP and CF from T1 through T3 were statistically significant in COVID-19 OU HCWs but they were not statistically significant in the control HCW group. No extra-systole and/or anomalies in the workers’ heart rhythms were detected.

**Figure 1:**
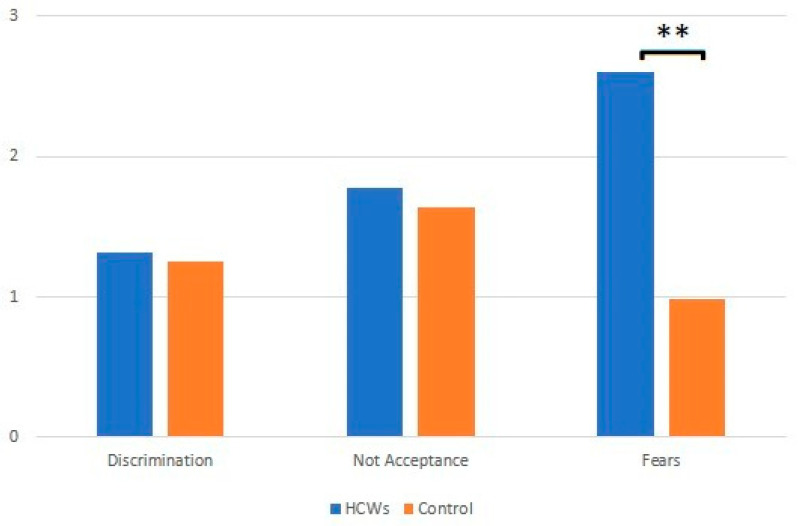
Results of SSPCS questionnaire (significance: ** *p* < 0.01).

From the analysis of SSPCS questionnaire answers given to HCWs working in COVID-19 wards, it is clear that all of them (both those working with COVID-19 patients and the controls) showed a sense of duty towards their patients. All HCWs reckoned that it was extremely important to use individual protection devices (IPDs) to prevent infections; compared to controls, a significantly higher number of HCWs caring for COVID-19 patients reported that it was important to use IPDs. Figure 1 reports a summary of the results obtained with the SSPCS questionnaire.

**Figure 2:**
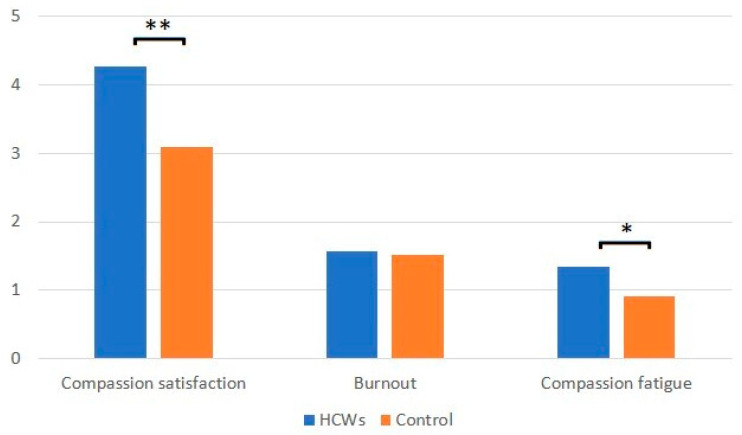
Results of ProQOL questionnaire (significance: * *p* < 0.1, ** *p* < 0.01).

Among HCWs working in COVID-19 OUs, the prevailing attitude was fear (2.8 vs. 0.98), with a statistically significant difference compared to controls; however, HCWs did not absolutely discriminate against those who had COVID-19 nor did they refuse to help those in need (discrimination 1.32 vs. 1.25). There was, however, considerable resistance in acceptance (1.78 vs. 1.64). Figure 2 reports the summary of the ProQOL questionnaire.

CS is the prevailing attitude in the answers and it is remarkably greater in HCWs working in COVID-19 OUs than in the controls. It also includes the agreeable sensation of helping others through one’s work, which is the positive side of the nursing professions, as well as the satisfaction that comes from feeling competent and able to carry out the job.

If we analyze the ProQOL answers, it is evident that there is great satisfaction and pride in being able to help patients without being overwhelmed by fatigue and fear (CF: COVID-OU HCWs vs. control HCWs). Pressure, stress and the related feelings may trigger sensations of powerlessness and inadequacy in one’s work. It is therefore important to understand what one can really do to help others. The BO dimension showed no statistically significant differences in either group.

## 4. Discussion

The new 2019 coronavirus or SARS-CoV-2 has been the first biological agent to generate, in this millennium, such a global health emergency as to determine the adoption of public health measures, such as social distancing, work limitations and smart working worldwide [3,37,38].

Major studies carried out in China, Italy and the USA have claimed that the most critical issues related to virus spreading and transmission have been impacting hospitals and, in particular, HCWs who, so far, still represent the professionals with the highest risks of infection [4]. The quick transmission of the disease and the growing number of new cases and deaths have brought about a remarkable state of anxiety and fear [37,39]. HCWs were exposed to significant stress, mainly due to direct treatment of COVID-19 patients, increased risk of infection, the fear of transmitting it to their own families, the growing concern for themselves and their loved ones, and often feeling stigmatized and secluded by others owing to fear of infection [37,39].

In addition, the growing number of cases and disease-related deaths, the heavy workload for long hours and IPDs being out of stock, especially in the early phase of the pandemic are all factors that have over time triggered emotional changes, which also impacted the workers’ physical conditions [4,6,40].

The purpose of the present study was to assess the cardiocirculatory functionality of a group of HCWs working in COVID-19 wards. A few psychological variables have been evaluated in order to assess perception of stress while at work.

All HCWs, in compliance with current legislation, were periodically tested with a molecular swab for SARS-CoV-2, with a surveillance plan aimed to protect all employees, depending on specific risks. All of them were duly vaccinated with two doses of the Comirnaty vaccine (BioNTech, Pfizer), administered in Jan–Feb 2021. Indeed, being vaccinated was mandatory to be able to work in areas with higher SARS-CoV-2 infection risks, as provided by the Italian legislation.

Cardiocirculatory parameters were analyzed at three different moments: 2 h before the shift (T1); throughout the whole 4 h shift (T2); and 2 h after the shift (T3). At T1, the ECGs of all HCWs were normal; similarly, mean basal values of SAP, DAP and CF (T1) fell within normal ranges in both groups under examination [36]. The results obtained by analyzing HoC and Hop instead showed significant variations in the cardiocirculatory activity. Particularly, mean values of SAP and DAP recorded at T1 were significantly higher at T2 and went back to basal levels at T3, although in a statistically significant way. CF values recorded at T1 were significantly higher at T2, then returned to basal levels at T3 in a statistically significant way. These differences were statistically significant also when compared to the control group. These results were also confirmed by the statistical analysis that compared the variables for all three time points. Indeed, mean values of SAP, DAP and CF from T1 through T3 were statistically significant in COVID-19 OU HCWs but they were not statistically significant in the control HCW group.

This is the first study where the SAP, DAP and CF of HCWs working in COVID-19 wards have been assessed; most studies on HCWs have mainly investigated the psychological impact related to the stress perceived while working in this specific context [10,39,41,42,43,44,45,46,47,48,49,50].

The pathophysiological mechanism that may have brought about changes in cardiocirculatory activity might be attributable to a greater stimulation, especially in stressed subjects, of the sympathetic system, which plays a crucial role in the control of blood pressure and vessel narrowing (fondazioneveronesi.it). Indeed, physical, psychological and social stimuli (stressor events) may cause hypothalamic secretion of corticotropin hormone (CHR) and a stimulation of the adrenal medulla, with an increase in catecholamines (adrenaline and noradrenaline). The main stress-responding hormonal mediator is the hypothalamic–pituitary–adrenal axis: in fact, under stressful conditions, cortical and subcortical centers control the activation of the hypothalamic paraventricular nucleolus, which triggers a neuroendocrinal reaction and is crucial to maintaining homeostasis [23,25,26,31].

Epidemiological data shows that chronic stress foretells the onset of coronary disease (CHD). Workers suffering from work-related stress and socially isolated or lonely persons run greater risks of a first coronary disease event [23,25,26,31]. Furthermore, short-term emotional stress can act as a triggering factor of cardiac events among individuals with advanced atherosclerosis [23,25,26,31].

In this study, workers were 40 years old on average, with no evident pre-existing cardiovascular illness. Some clinical guidelines consider stress a prevention target for people with high overall risks of cardiovascular diseases or with known CVDs [25].

Our results are in line with what is reported in literature [10,17,39,51]. The persistence of working conditions perceived as stressful may, in the long run, bring changes to the cardiocirculatory system which, in more susceptible subjects, might end up becoming real pathologies.

By analyzing the answers to the SSPCS questionnaire, it is evident that all HCWs showed an excellent sense of duty towards their patients, both those dealing with COVID-19 subjects and the control group. All this is in line with the scientific literature which highlights that despite being stigmatized, HCWs never fail to show a high sense of responsibility and dedication to their patients [10,17,39,51]. HCWs’ answers emphasized the importance of using IPDs to prevent infections, especially those workers engaged in assisting COVID-19 patients relative to controls. To the question “Even just to talk to someone COVID-19 infected, would you wear a mask to prevent infection?”, the answer was “I definitely would”. In this study, the prevailing feeling was that of fear, whereas the least frequent was discrimination, as is observable in Chart 1.

Data from the literature data suggests that COVID-19-induced levels of fear and HCWs’ fatigue diminished more and more over time, whilst satisfaction slightly increased [16,18,52].

High levels of COVID-19-related fear were identified in individuals with chronic diseases—a predictable result, as all the sources of information highlighted that COVID-19 mostly affects people with chronic health problems. This type of information may obviously have played a role in raising fears of COVID-19 in individuals with such problems [41]. A survey by Bakioğlu et al. (2021) [41] showed that fear of COVID-19 has a positive relationship with anxiety, depression and stress [41]. While fear, within certain limits, is thought to be useful to motivate people to effectively respond to a given threat or stimulus, extreme and persistent fear may generate negative psychological reactions like stress, depression and anxiety [42,43,53].

By analyzing the PorQOL results, it is clear from answers that there is great satisfaction and pride in being able to help one’s own patients without giving in to fatigue and fear. Emotional pressure, stress and a combination of these feelings can trigger a sense of powerlessness and inadequacy in one’s own work [42,43,44,45,46,53].

It is therefore important to acknowledge what one can really do to help other fellow individuals. Besides, this did not lead to the devaluation of one’s own person or to the loss of confidence in oneself, such that HCWs maintained good self-esteem and did not have negative thoughts about themselves [42,43,44,45,46,53].

CS has been the prevailing dimension in our study, which involves the positive side of care-related jobs, and the satisfaction deriving from feeling competent and being able to carry out the job well. It also includes the pleasant sensation of helping others with one’s job and that is exactly what emerges from this study [43,44,45,46].

In a comparative study of a group of HCWs in close contact with COVID-19 patients compared to control HCWs, Wu et al. (2020) [42] concluded that the rate of mental disturbances (i.e., burnout, depression, post-traumatic stress disorder) was significantly higher [42]. Huang et al. (2020) [53], in their assessment of front line HCWs, reported a 27.39% prevalence of stress disorder, with this prevalence being higher in women. They noted the high prevalence of these disorders among front-line HCWs and highlighted the need to seriously take mental health and psychological skills training into account [53]. These outcomes align with those obtained in our survey based on the CF-related questions.

CF (literally “compassionate fatigue”) is a condition characterized by a gradual and progressive reduction of the willingness to take care of others, that is to say, compassion [43,44,45,46].

BO is a set of symptoms deriving from a chronic, persistent stress condition linked to the work context. Someone who suffers from it gets to the point of feeling like “I can’t go on like this anymore” and feels totally dissatisfied and distraught by their daily routine [43,44,45,46]. In this study, questions like “Did I feel exhausted due to my job?” or “Was I overwhelmed by the work carried out?”, which dealt with the BO dimension, were most frequently answered with “sometimes” or “often”, which is a sign of the high fatigue experienced by HCWs, both those working in COVID-19 wards and the controls.

Managing stress and caring about one’s mental health is crucial to maintaining physical health and includes organizing one’s work as best as one can; working a reasonable number of hours and taking breaks; and connecting with colleagues, which is fundamental to the coordination of activities, the sharing of personal perceptions and to finding reciprocated support, and is also related to respecting the different ways to react to critical situations [12,16,18,54,55,56,57]. Emotional pressure, stress and a combination of these feelings can arouse a sense of powerlessness and inadequacy towards one’s own job. It is therefore important to realize what one can really do to help others by appreciating even little positive achievements; thinking about what turned out well and accepting what did not live up to expectations; and recognizing circumstance-related limitations [46,58,59].

In a study dealing with HCWs working in COVID-19 high-risk areas, Lu et al. (2020) [60] observed that levels of anxiety and depression were higher in these workers than in others working in low risk hospitals [60], which is in line with our results. Ni et al. (2020) [61] carried out a survey of 214 HCWs and found that anxiety and depression rates were higher than those in the general population [61]. Another study conducted by De Los Santos and Labrague (2020) [62] showed that 37.8% of front-line nurses experienced dysfunctional levels of anxiety related to the COVID-19 pandemic [62].

The weaknesses of this study are the sample size and the short period of observation, which did not allow us to draw definitive conclusions. In fact, a more conspicuous sample and, above all, a longer period of observation would allow for a deeper knowledge of which factors may have triggered the cardiocirculatory changes detected.

The strength of this study is that for the first time the direct and indirect effects triggered by the “COVID-19 phenomenon” have been addressed, as we have evaluated how this phenomenon has worked organically and systemically, but especially on a cardiovascular as well as physical level.

## 5. Conclusions

The results obtained prove that there were significant changes in arterial pressure and CF while HCWs provided assistance in COVID-19 wards. This stems from a probable psychological and physical state of stress, even though the sense of duty and the willingness to help others remain unaltered, as is clearly deduced from the answers to the two questionnaires used.

Particular attention should be paid to the observed CV changes, since there may be several problems related to them. Indeed, hypertension and high cardiac frequency may be major risk factors; in other words, they may be conditions which increase the likelihood of CV diseases, such as angina pectoris, myocardial infarction, and brain stroke until cardiac arrest.

It is important to pay attention to HCWs’ mental health, especially in the contexts of stress and anxiety, since these workers are entrusted with the delicate task of lending assistance to patients [54,55,56,57].

Continuous monitoring of these employees, as well as occupational medicine surveillance, allows for the detection of critical conditions and for the implementation of actions aimed at preventing chronic processes from occurring, which might, in the long run, lead to real pathologies [54,55,56,57,63].

## Figures and Tables

**Table 1:** Main characteristics of the sample.

	COVID-19 OU 30 (100%)	No COVID-19 OU 30 (100%)	*p*-Value
Gender (men)	18 (60%)	18 (60%)	n.s.
Gender (women)	12 (40%)	12 (40%)	n.s.
Mean age (yrs)	41.1 ± 7.7	40.3 ± 8.1	n.s.
Working seniority (yrs)	10.5 ± 6.4	10.1 ± 5.6	n.s.
BMI	24.7 ± 2.8	24.4 ± 6.7	n.s.
Non-smoker	30 (100%)	30 (100%)	n.s.
Family history of cardiovascular diseases	5 (17%)	6 (20%)	n.s.
Anti-COVID-19 Vaccination	30 (100%)	30 (100%)	n.s.
Physicians	9 (30%)	10 (33%)	n.s.
Nurses	15 (50%)	15 (50%)	n.s.
Healthcare assistants	6 (20%)	5 (17%)	n.s.
Shift work	30 (100%)	30 (100%)	n.s.
Full-time worker	30 (100%)	30 (100%)	n.s.
Permanent contract	18 (60%)	17 (57%)	n.s.
Fixed-term contract	12 (40%)	13 (43%)	n.s.

n.s.: not significant.

**Table 2:** Mean values of SAP, DAP and CF in COVID-19 OU HCWs and in the control group, detected during 8 recording hours (T1–T3).

	T1		T2		T3	
	HCWs	Control	HCWs	Control	HCWs	Control
SAP (mmHg)	120.2 ± 8.7 *^#^	119.3 ± 9.8	149.1 ± 11.8 *^°ç#^	122.4 ± 9.7 ^ç^	128.4 ± 15.7 ^°#^	121.9 ± 10.4 ^ç^
DAP (mmHg)	80.6 ± 114 *^#^	80.5 ± 7.2	103.7 ± 32,7 *^°ç#^	82.6 ± 8.4 ^ç^	86.6 ± 7.3 ^°#^	82.3 ± 6.1 ^ç^
CF (bpm)	79.8 ± 6.0 *^#^	78.6 ± 4.7	104.6 ± 13.7 *^°ç#^	79.8 ± 7.2 ^ç^	90.7 ± 16.5 ^°#^	80.7 ± 6.6 ^ç^

HCWs: Healthcare Workers; *p*-value < 0.05: * T1 vs. T2; ° T2 vs. T3; ^ç^ HCWs vs. Controls; ^#^ T1 vs. T2 vs. T3.

## Data Availability

Not applicable.
